# Electroactive microneedle augmented stem cell therapy in myocardial infarction

**DOI:** 10.1126/sciadv.aeb4840

**Published:** 2026-05-06

**Authors:** Wentao Zhang, Yiqi Shen, Shuangxu Jia, Yingxin Chen, Ruisi Cai, Yunlong Jiao, Quanquan Han, Jiahuan You, Shiqi Wang, Xingyi Wan, Jicheng Yu, Zhen Gu, Zhuxiao Gu, Yuqi Zhang

**Affiliations:** ^1^ State Key Laboratory of Advanced Drug Delivery and Release Systems, School of Pharmacy, Zhejiang University, Hangzhou 310058, China.; ^2^ Jinhua Institute of Zhejiang University, Jinhua 321299, China.; ^3^ College of Materials and Environmental Engineering, Hangzhou Dianzi University, Hangzhou 310018, China.; ^4^ Department of General Surgery, Sir Run Run Shaw Hospital, School of Medicine, Zhejiang University, Hangzhou 310016, China.; ^5^ Liangzhu Laboratory, Zhejiang University, Hangzhou 311121, China.; ^6^ MOE Key Laboratory of Macromolecular Synthesis and Functionalization, Department of Polymer Science and Engineering, Zhejiang University, Hangzhou 310027, China.; ^7^ Institute of Fundamental and Transdisciplinary Research, Zhejiang University, Hangzhou 310027, China.; ^8^ Engineering Research Center of Innovative Anticancer Drug, Ministry of Education, Hangzhou 310058, China.; ^9^ Key Laboratory of Modern Acoustics (MOE), Department of Physics, Nanjing University, Nanjing 210093, China.; ^10^ Department of Burns and Wound Care Center, Second Affiliated Hospital, School of Medicine, Zhejiang University, Hangzhou 310009, China.

## Abstract

Ischemic heart disease and related sequelae pose tremendous burdens on worldwide medical care. The excessive activation of cardiomyocytes and cardiac fibroblasts further exacerbates the prognosis after necrosis. Decades of stem cell therapy in preclinical studies suggested promising results in cardiomyocyte regeneration and tissue remodeling. However, few formulations achieved clinical translation due to the limited stem cell engraftment and insufficient arousal of resident cardiomyocytes. Here, we reported an implantable electroactive device to leverage stem cell therapy and cardiomyocyte restoration for effective heart recovery. Assisted by the piezoelectric microneedle patch with 80–cubic millimeter cavity, 1.5 × 10^5^ mesenchymal stem cells could be delivered efficiently to the infarcted site and sustained longer for continuous paracrine effects. Meanwhile, the piezoelectric stimulation generated from the poly(l-lactic) acid microneedle matrix further potentiated the stem cells and elicited more vigorous self-repair responses in cardiomyocytes. This approach was validated to effectively suppress inflammatory monocytes, reduce cardiomyocyte necrosis, and improve heart remodeling in a rat heart infarction model.

## Teaser

Microneedle-generated electrical stimulation potentiates stem cells and promotes cardiac regeneration.

## INTRODUCTION

Ischemic heart disease (IHD) is a leading cardiac emergency that affects millions of individuals worldwide (*1*). Its pathogenesis is primarily driven by incident rupture or gradual erosion of atherosclerotic coronary plaque, which exposes the highly thrombogenic core to the bloodstream and subsequently triggers the coagulation cascade. One direct consequence is cardiomyocyte necrosis at the ischemic region that can hardly be self-restored without medical interventions such as pharmacological treatments and reperfusion strategies (*2*). However, these conventional procedures fail to recover the injured cardiomyocytes, and subsequent signaling cascades further exacerbate the heart failure through maladaptive tissue remodeling (*3*). In response to ischemic stress, the infarcted heart activates cardiac fibroblasts and remodels cardiomyocytes, which often leads to excessive fibrogenesis and hypertrophy, further deteriorating cardiac function (*4*). Therefore, an effective therapeutic approach is urgent to address the cascade of post–myocardial infarction events, from modulating immune cell infiltration to promoting self-resolution processes.

Regenerative cell-based therapies have achieved remarkable advancements in tissue engineering over the past decades, offering potential strategies for cardiac repair (*5*–*8*). However, in terms of heart regeneration, major challenges remain, including the poor engraftment of transplanted cells, as well as inadequate interactive communication between transplanted and endogenous cells (*9*, *10*). Delivery strategies have been explored in the forms of vesicles (*11*–*13*), cell sheets (*14*–*16*), hydrogels (*17*–*19*), and cardiac patches (*20*–*22*) to improve the survival and persistence of transplanted stem cells. Our previously developed perforated microneedle patch demonstrated exceptional potential in localized cell delivery with substantial loading capacity, efficient tissue penetration, and well-preserved cell viability (*23*). Nonetheless, optimizing the therapeutic functionality of stem cells and fostering effective interactions between transplanted cells and surviving cardiomyocytes at the infarcted region remain critical to fully harnessing the potential of stem cell therapy for heart regeneration.

**Fig. 1.**
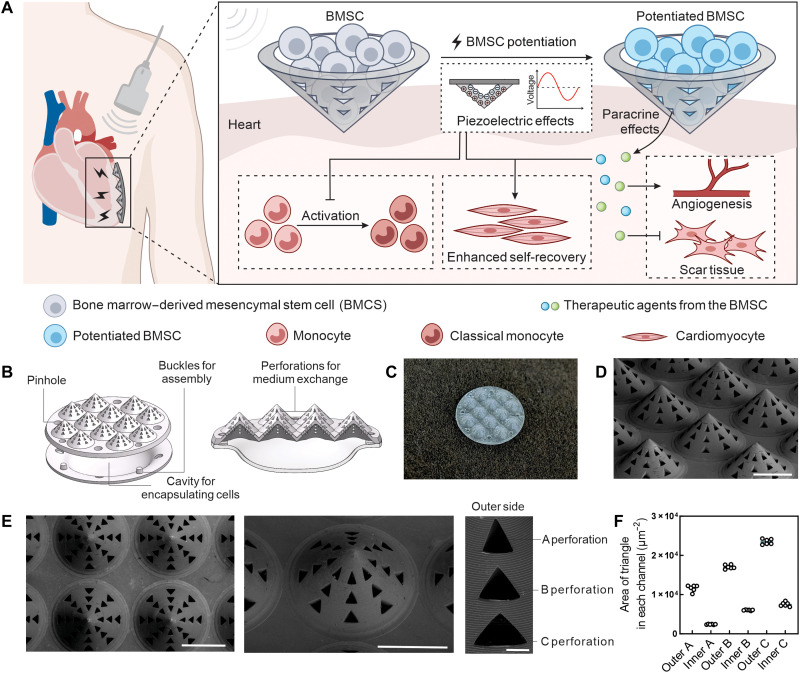
Design of the piezoelectric IEMP delivering BMSC for cell therapy of MI. (**A**) Scheme of piezoelectric IEMP-mediated stem cell therapy to potentiate BMSC, inhibit classical monocyte activation, enhance the self-recovery of cardiomyocytes, promote angiogenesis, and reduce scar tissue. (**B**) The 3D illustration of the structure and assembly of IEMP and the base. (**C**) The photograph of IEMP. (**D** and **E**) Representative scanning electron microscopy (SEM) images of the IEMP. Scale bar, 1000 μm for the macro-view and 100 μm for the zoom-in. (**F**) The measured area of the triangular shape of each perforation from the outer and inner sides based on the SEM images. *n* = 6 from independent needle tips.

Here, we boosted the therapeutic potential of transplanted bone marrow–derived mesenchymal stem cells (BMSCs) using an implantable electroactive microneedle patch (IEMP). This bioengineered platform could maintain high cell viability and functionality in localized cell delivery for acute myocardial infarction (MI), meanwhile promoting cellular cross-talk and self-regenerative potency through piezoelectric stimulation. The IEMP was fabricated from a piezoelectric polymer by a “supercooling molding” technique, featuring micro-sized channels on the shell. After implantation, the IEMP could effectively anchor a substantial quantity of BMSCs with minimal damage and longer resident time to the infarcted site. The precisely designed channels ensured robust paracrine effects and prompt communication between implanted BMSCs and endogenous cardiomyocytes. Furthermore, the IEMP matrix, made from poly(l-lactic) acid (PLLA), generates piezoelectric stimulation to embedded BMSCs and adjacent cardiomyocytes through noninvasive ultrasonic actuation. The electrical stimulation and piezoelectricity previously reported in restoring normal electrophysiological function were presently found as an efficient stimulus in modulating cells behaviors (*24*, *25*), boosting BMSC proliferation (*26*), and accelerating tissue regeneration (*27*–*30*). The presence of piezoelectricity from IEMP substantially improved the paracrine of BMSCs and elicited the self-recovery of cardiomyocytes. In the rat MI model, the IEMP-potentiated BMSCs functioned as an endurable therapeutic medic, extending their paracrine activity, promoting angiogenesis, and inhibiting the generation of scar tissue (Fig. 1A). Moreover, the in situ–generated piezoelectric stimulation modulated the immune response by rebalancing the classical and nonclassical monocyte populations at the acute inflammation stage.

## RESULTS

### Fabrication of the IEMP device

IHD led to devastating necrosis of cardiomyocytes and aberrant adaptive remodeling with impaired electrical function. Electric stimulation, traditionally used to restore normal electrophysiological activity after cardiac disorders, such as cardioverter defibrillators and pacemakers, has presently emerged as a promising strategy to modulate cardiomyocyte differentiation and prognosis after stem cell transplantation for tissue regeneration (*24*, *31*, *32*). In this study, we incorporated the electricity-generating moiety into the stem cell delivery system by an IEMP device to potentiate the regenerative capacity. The IEMP was fabricated from a biodegradable polymer, PLLA, by a supercooling molding method. PLLA has been reported as a potent piezoelectric material that can convert mechanical energy to electrical stimulation in various biomedical applications, such as biosensing and tissue regeneration (*27*, *33*–*37*). Conventional approaches such as melt stretching, thermal annealing, and electrospinning can efficiently induce the piezoelectric performance of PLLA (*33*, *38*, *39*); however, they are not feasible to generate dedicated geometries such as microneedles, capsules, or structural frames.

We here explored a fabrication approach, the supercooling molding method, which enabled the precise customization of PLLA into a diverse range of shapes. Specifically, this process involved first melting the PLLA at a high temperature (235°C), followed by cooling it below its equilibrium melting temperature (157°C) with a three-dimensionally (3D) printed mold. This isothermal incubation for 2 hours initiated controlled crystallization of PLLA, increasing the portion of the β phase responsible for enhanced piezoelectric performance. Using the above technology, we were able to obtain the piezoelectric IEMP device with complex geometries for stem cell delivery. The IEMP was customized into 12 blunt needle tips with a large cell encapsulating cavity of around 80 mm^3^ and 24 channels on each tip for medium exchanges (Fig. 1B). This microneedle configuration avoided direct tissue penetration but instead maximized the cavity space and tissue-material interface. A round base with a diameter of 9 mm was affiliated to prevent the cell leakage or rubbing by the chest interior wall. The 24 extrusion parts on the polydimethylsiloxane (PDMS) bottom mold lifted the top mold and perforated the molten PLLA to form 24 perforations (Fig. 1, C and D, and fig. S1). The size of each tip was 1 mm in height, 1 mm in radius, and 2 mm in center-to-center distance (Fig. 1E). The measured area of the inner side of these perforations ranged from 2.40 × 10^3^ to 7.52 × 10^3^ μm^2^, and that of the outer side ranged from 1.16 × 10^4^ to 2.35 × 10^4^ μm^2^ (Fig. 1F). These 24 perforations on each tip therefore granted sufficient exchanging tunnels between implanted BMSCs and resident cells.

### The piezoelectric performance of the IEMP

**Fig. 2.**
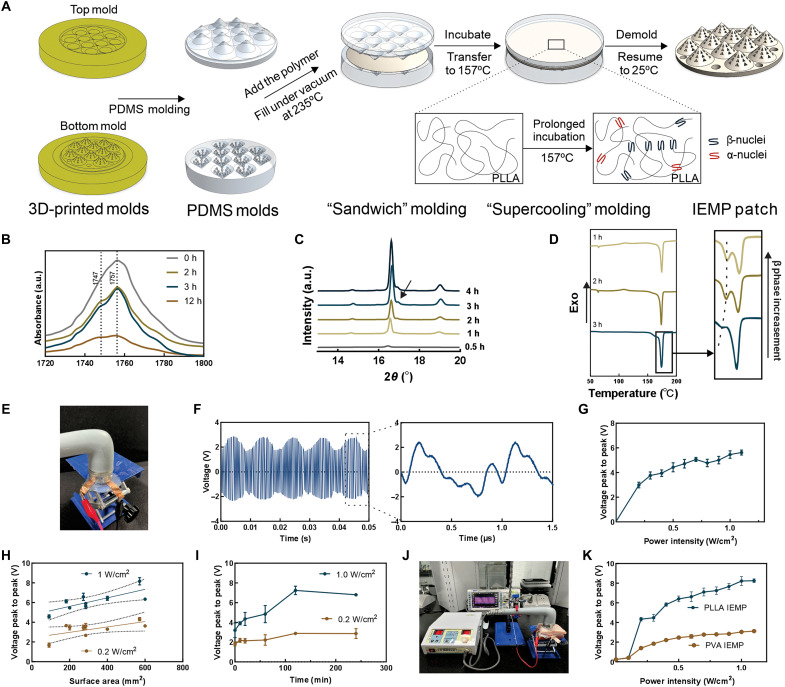
The manufacture and characterizations of the piezoelectric IEMP device. (**A**) Schematic presenting the fabrication process of the IEMP patch. (**B**) Fourier transform infrared spectra were measured for the samples with different treating durations of supercooling. Lines at 1747 and 1757 cm^−1^ mark the characteristic band. (**C**) The 1D-wide-angle x-ray diffraction (WAXD) patterns of samples with various times of supercooling. The black arrow marks the presence of the β phase. (**D**) The DSC curves for samples with different times of supercooling. The curves depicting the scenarios when β phase further increases were graphic mimics from . (**E**) The photograph of the in vitro measurement device for piezoelectricity. (**F**) The elicited piezoelectricity (and zoom-in) by an ultrasonic machine at the intensity of 0.5 W/cm^2^. (**G**) The voltage outputs at different power intensities. *n* = 3 from independent samples. (**H**) The voltage outputs of PLLA films with different surface areas after different times of supercooling. *n* = 3 from independent encapsulation and measurement. Simple linear regression was conducted by Prism 9.0.0 with 95% confidence bands. (**I**) The voltage outputs at different times of supercooling. *n* = 3 from independent samples. (**J**) Photograph of the ex vivo experimental setup under the porcine skin. (**K**) The voltage outputs at different power intensities in ex vivo setup. *n* = 5 from independent samples. Data are means ± SD. h, hours. a.u., arbitrary units.

The IEMP was fabricated through a self-developed supercooling molding method assisted with two 3D-printed molds (Fig. 2A). The supercooling-treated PLLA exhibited two characteristic bands at 1747 and 1757 cm^−1^ in the Fourier transform infrared spectra, indicating the dipole alignment and phase transition along with the incrementing time (Fig. 2B) (*40*). The x-ray diffraction (XRD) results gave details of this transition that partial nuclei managed to be β nuclei, and the β phase grew extensively with prolonged treating time (Fig. 2C). The formation of the β phase also affected the melting behavior of PLLA, as shown in differential scanning calorimetry (DSC) curves, wherein it slightly elevated to around 166°C (Fig. 2D). This temperature elevation could become more pronounced if the β phase became increasingly dominant as indicated by previous report (*39*). To be noted, the PLLA polymer did not align as the previous melt-stretching method did, and the β phase detected by XRD and DSC represented its scattered distribution on the surface. Therefore, its piezoelectric property varied at different regions but potentially responded to all stress from all directions. The piezoelectric coefficient measured around 6.54 pC/N after supercooling for 12 hours was sufficient to generate piezoelectricity, although its theoretical capacity was lower than the aligned PLLA film (fig. S2).

To accurately measure the piezoelectric performance of PLLA, the IEMP or the PLLA film was sealed by silicone to prevent external interference (Fig. 2E). The piezoelectric IEMP with an area of 63.6 mm^2^ (9 mm in diameter) was able to generate stable electrical outputs around 4.6 V under a low ultrasound intensity (0.5 W/cm^2^) (Fig. 2F). The increase of ultrasonic intensity up to 1.1 W/cm^2^ further augmented the voltage output to 5.6 ± 0.2 V (Fig. 2G). The voltage generation could be further enlarged by increasing the surface area to capture the energy from the ultrasound probe (9.62 × 10^2^ mm^2^) (Fig. 2H). In addition, the cumulative effects of the β phase played vital roles in strengthening the overall piezoelectric performance. The prolonged supercooling procedure substantially improved the piezoelectricity of the IEMP device (Fig. 2I). However, excessive treatment produced increased crystallization, which would compromise the mechanical integrity by exacerbating its brittleness and diminishing its ability to maintain structural stability (fig. S3). Therefore, a supercooling duration of 2 hours was determined to prepare the device, balancing the electrical performance and mechanical property.

To further verify the dominating effects of piezoelectricity, we examined the performance of the IEMP in an ex vivo setup on porcine skin (Fig. 2J). The nonpiezoelectric polyvinyl alcohol (PVA) was chosen as a negative control to fabricate the microneedle patch with identical geometry to IEMP. Therefore, both patches exhibited comparable triboelectric potential, allowing for a more precise assessment of piezoelectric contributions. The PVA-based microneedle patch suffered a remarkable reduction in voltage output due to the cushioning effect within the tissue. In contrast, the PLLA-based IEMP even generated higher voltage output than in the in vitro setting. This enhancement could be attributed to the more efficient transmission of ultrasound energy through biological tissue (Fig. 2K).

### Loading of BMSCs into the IEMP with large quantity and high viability

**Fig. 3.**
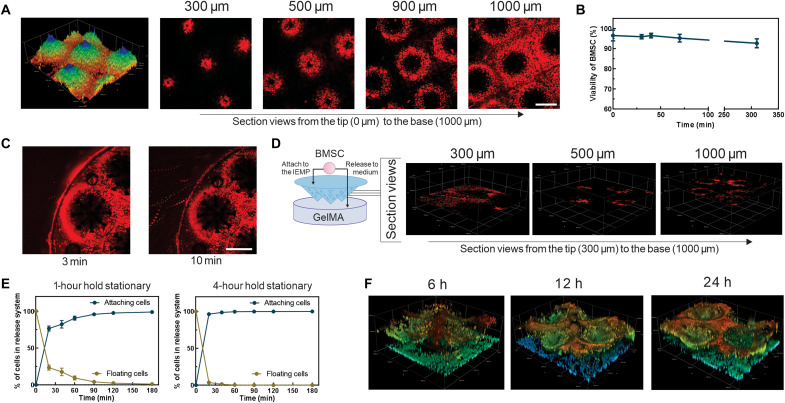
The BMSC-loading capacity of the IEMP device. (**A**) The 3D construction and section views of IEMP loaded with Dil-labeled BMSCs by confocal microscopy. Scale bar, 1000 μm. (**B**) The viability of BMSCs during the loading process. *n* = 4 from independent samples. (**C**) The overlapping images over 3 min (images were taken at 10-s time interval) after immersing the IEMP into the culture medium. Scale bar, 1000 μm. (**D**) The illustration of loaded BMSCs behavior into GelMA and the section views of representative layers after 6 hours (h) of release onset. (**E**) The percentages of attaching and floating cells in the IEMP device after 1 or 4 hours holding stationary after the cell loading. *n* = 10 from independent samples. The attached cells were calculated on the basis of the floating cells. (**F**) The 3D construction of IEMP loaded with Dil-labeled BMSCs after different time points of release.

Next, the feasibility of loading BMSCs into the IEMP device was assessed. The generated PLLA exhibited decent biocompatibility with cardiomyocytes and BMSCs (fig. S4). For cell loading, 1.5 × 10^5^ BMSCs in 50 μl of 4% gelatin DMEM (Dulbecco’s modified Eagle’s medium) were injected into the cavity of the IEMP. The confocal images confirmed that Dil-labeled BMSCs occupied the whole needles from the tip to the base (Fig. 3A). Besides, the loading procedures exhibited negligible impairment on cell viability after 6 hours of residing in the IEMP (92.6 ± 2.2%) (Fig. 3B). There was no obvious cell leakage observed from the assembled device after being immersed in the medium for 10 min, suggesting the ability of IEMP to constrain the stem cells. (Fig. 3C).

We further investigated the release behavior of BMSCs from the IEMP into a gelatin methacrylate (GelMA) hydrogel medium. If BMSCs were loaded without fixation, the solidified gelatin gel in IEMP would liquefy under the physiological condition, and the embedded BMSCs would migrate across the perforations into the medium swiftly (Fig. 3D). This phenomenon demonstrated a well-connected passage between IEMP inside and outside. However, to avoid the cell drifting and leakage after IEMP implantation, we leveraged the intrinsic fibroblastic properties of BMSCs, allowing them to adhere spontaneously to the interior surface of the IEMP. Quantitative analysis revealed that holding the IEMP stationary for 1 hour postloading resulted in 76.5 ± 3.4% cell attachment within 20 min of release onset. Extending the stationary period to 4 hours further increased cell attachment to around 96.4% (Fig. 3E). A markedly denser BMSC layer was observed 24 hours after incubating in a GelMA gel, suggesting an active cellular spreading and colonization within the IEMP (Fig. 3F).

### IEMP-generated piezoelectric stimulation magnified the therapeutic potential of BMSCs

**Fig. 4.**
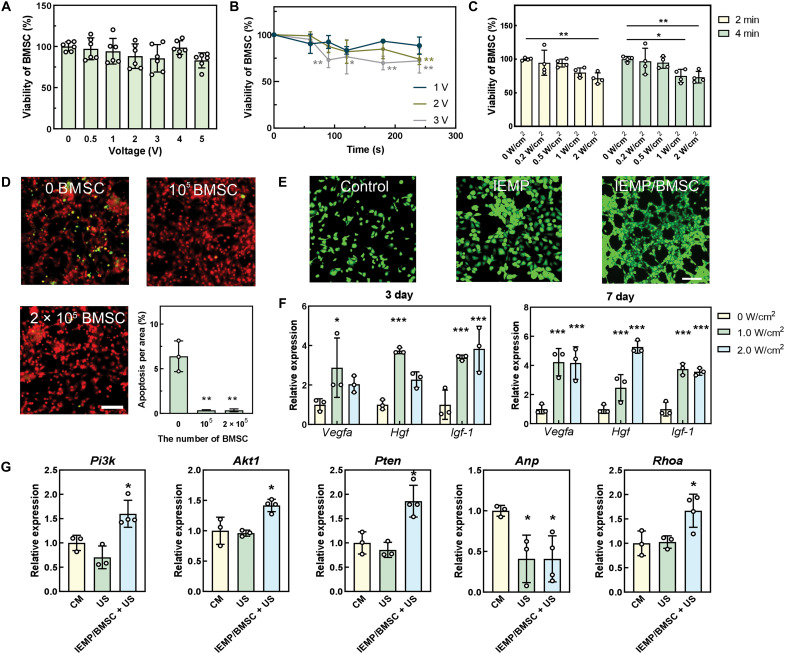
The strengthened effects of piezoelectric stimulation on cardiomyocyte survival. (**A**) The viability of BMSCs after stimulation at different voltages (AC, alternating current) for 2 min. *n* = 6. (**B**) The viability of BMSCs after stimulation at different voltages for different times. *n* = 3. (**C**) The viability of BMSCs after stimulation by IEMP and different intensities of ultrasound. *n* = 4. (**D**) The fluorescent images showing cardiomyocyte apoptosis (green) after incubation with different amounts of BMSCs for 24 hours. The quantitative analysis was based on the images. Scale bar, 100 μm. *n* = 3. (**E**) The angiogenesis assay presenting the tube formation after treating with IEMP/BMSC or IEMP. Scale bar, 100 μm. (**F**) The relative expressions of *Vegfa*, *Hgf*, and *Igf-1* compared to *Gapdh* after stimulation with IEMP and ultrasound for 3 days. *n* = 3. (**G**) Relative expressions of *Pi3k*, *Akt1*, *Pten*, *Anp*, and *Rhoa* after treating cardiomyocytes (CM) with IEMP/BMSC + ultrasonic actuation or ultrasonic actuation only. Data are means ± SD *P < 0.05; **P < 0.01; ***P < 0.001.

Beyond confirming cell survival, we further investigated the influence of piezoelectric stimulation from the IEMP on BMSCs and cardiomyocytes. BMSCs demonstrated sustained viability after the application of external electric fields for 2 min (Fig. 4A). Only a portion of BMSCs lost viability after extended exposure to electric stimulation (Fig. 4B). While stimulated by IEMP with ultrasound, most of the cells withstood the increment of ultrasonic power intensity and generated piezoelectricity (Fig. 4C). The same magnitude of ultrasonic power and generated voltage had limited impact on cardiomyocyte viability but did increase the beating rate (fig. S5).

The cardiomyocytes had limited regenerative potential and suffered from severe impairment after ischemic stress. The presence of BMSCs significantly reduced the apoptosis rate of cardiomyocytes under hypoxic conditions, which could primarily be attributed to paracrine factors released by these stem cells (Fig. 4D). The tube formation of human umbilical vein endothelial cells also suggested that BMSCs in IEMP equipment had an angiogenetic potential, whereas solely piezoelectric effects from IEMP did not (Fig. 4E). Notably, the electrically stimulated BMSCs exhibited enhanced paracrine effects that promoted regenerative capacity. Following periodic piezoelectric stimulation for 3 days, significant elevations in mRNA levels among *Vegfa*, *Hgf*, and *Igf-1* were detected from BMSCs (Fig. 4F). These enhancements were even maintained for 7 days both in mRNA and protein levels, suggesting the long-term therapeutic potential of the trained BMSCs (Fig. 4F and fig. S6). Whereas solely ultrasound treatment to the BMSCs had no significant impact (fig. S7).

The collected therapeutic effects of BMSCs and piezoelectricity on cardiomyocytes were also revealed by mRNA expression (Fig. 4G). The cardiomyocytes under ischemic conditions were treated with BMSC-embedded IEMP and ultrasound. The results indicated that a phosphoinositide-3 kinase (*PI3K*)/serine/threonine protein kinase 1 (*Akt1*) signaling pathway, which is responsible for cell survival, was actively transcribed in the treated cardiomyocytes. Notably, this elevation was not aberrantly overactivated like the one in excessive fibrogenesis and hypertrophy because the inhibitor in this pathway, *Pten*, was also up-regulated, suggesting that cells were undergoing a highly controlled self-protection. Another key mRNA that participated in cell survival and remodeling, Ras homolog gene family member A (*Rhoa*), also exhibited elevated expression. Meanwhile, the atrial natriuretic peptide (*Anp*), which is often pathologically overexpressed after MI, was notably down-regulated, further supporting that the introduction of BMSCs and IEMP-mediated piezoelectricity elicited the regenerative potential of cardiomyocytes.

### IEMP-boosted cell therapy protected the heart from severe MI damage

**Fig. 5.**
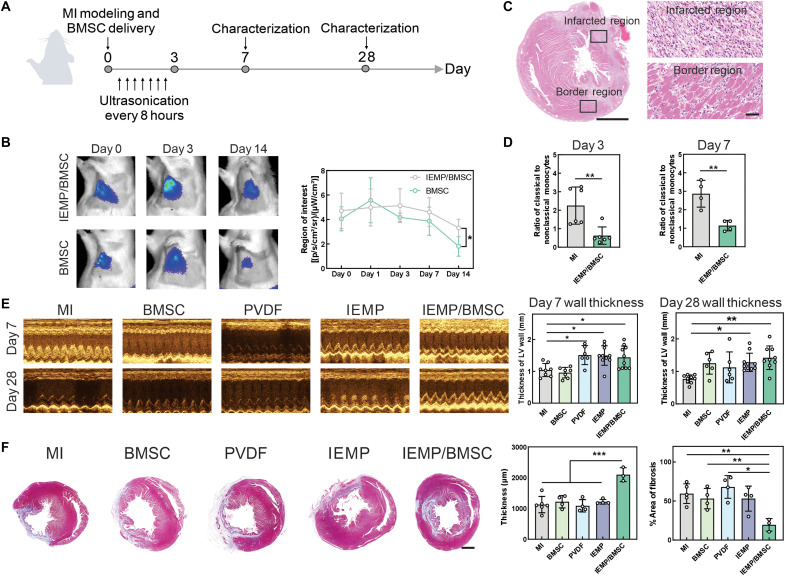
The thickening of hearts by IEMP-mediated BMSC therapy. (**A**) A timeline describing the ultrasonication treatment and characterization of the animal experiment. (**B**) Representative images showing the persistence of DiR-labeled BMSCs by IEMP/BMSC or intracardiac injection of BMSCs. The quantitative analysis was based on the images. *n* = 4. (**C**) Representative H&E staining of rat hearts on day 2. Scale bar, (left) 2 mm and (right) 50 μm. (**D**) Quantitative analysis of the ratio of classical monocytes (CD43_lo_His48_hi_) to the nonclassical monocytes (CD43_hi_His48_int-lo_) at days 3 and 7 based on the fluorescence-activated cell sorting results. (**E**) Representative M-mode echocardiography images at days 7 and 28 from different groups. The quantitative analysis was based on these images. *n* = 6 to 10 from independent individuals. (**F**) Representative Masson’s trichrome staining and quantitative analysis of wall thickness of the left ventricle based on the histological sections on day 28. Scale bar, 2 mm. *n* = 6 for the MI group and 3 to 4 for the others. Data are means ± SD *P < 0.05; **P < 0.01; ***P < 0.001.

Motivated by the protective effects on cardiomyocytes, we next evaluated the therapeutic performance of the BMSC-loaded IMEP on a rat acute MI model (Fig. 5A). Five groups were established as follows: MI group with no treatment, BMSC group with intracardiac injection of BMSCs, PVDF group with piezoelectric polyvinylidene fluoride-trifluoroethylene (PVDF-TrFE) film attached to the left ventricle as a positive control that effectively generated piezoelectricity under ultrasonication, IEMP group with blank IEMP attached to the left ventricle, and IEMP/BMSC group with BMSC-encapsulating IEMP attached to the left ventricle. The ultrasound was intensively applied to the anterior of the left chest on the initial 72 hours (once every 8 hours) to elicit piezoelectricity of the IEMP and potentiate the BMSCs. With the assistance of the IEMP, implanted BMSCs could reside longer than with intracardiac injection (Fig. 5B and fig. S8). Observed from the echocardiography, the BMSC group suffered a worse condition comparable to the untreated group, suggesting limited therapeutic efficacy from the intracardiac injected stem cells. While superior contraction abilities of hearts were perceived among the three piezoelectricity-generating groups (PVDF, IEMP, and IEMP/BMSC group) starting from day 3 (fig. S9). We therefore speculated that this phenomenon was attributed to the electrical stimulation generated from those piezoelectric materials (PVDF-TrFE and PLLA) to monocytes and macrophages. The influence of electrical stimulation on monocyte/macrophage polarization, migration, and phagocytosis has been reported in diverse diseases (*41*).

Previous literature have revealed the crucial participation of monocytes and macrophages in the pathology and repair process (*42*, *43*). In our investigation, massive monocytes cell infiltrations and collagen depositions were observed on day 2 in the hematoxylin and eosin (H&E) staining images of MI rats (Fig. 5C). At this stage, the dominating infiltrated immune cells were monocytes or transitioning from monocytes to macrophages. The fluorescent images of the MI rats on day 2 further excluded the presence of conventional CD86 marker of inflammatory macrophages in the examined regions of the heart tissue, a similar phenomenon described in the MI mouse model where monocytes dominated on day 3 and inflammatory macrophages peaked later on day 7 (fig. S10) (*44*–*46*). Also, extending period of piezoelectric stimulation from 3 to 14 days did not further increase the therapeutic outcome in our setting (fig. S11). These findings together implied that the primary modulatory effect of electrical stimulation in regulating disease progression may involve macrophage precursors, specifically the monocytes.

To further elucidate the impact of electrical stimulation on monocyte subpopulations, we used two recently identified markers, His48 and CD43, to distinguish between classical (inflammatory) and nonclassical (anti-inflammatory) monocytes (*47*). Flow cytometry analysis of bone marrow–derived macrophages demonstrated that exposure to 0.5-V voltage significantly reduced the ratio of classical monocytes to nonclassical monocytes (fig. S12). This trend also manifested in rats treated with IEMP/BMSC on days 3 and 7 (Fig. 5D). The introduction of IEMP/BMSC to the rat heart remarkably decreased the proportion of inflammatory monocytes, potentially mitigating collateral damage in the peri-infarct region.

Despite this immunomodulatory effect, electrical stimulation alone was insufficient to rescue the ischemic myocardium, as evidenced by impaired contraction of the anterior left ventricular wall from day 7 onward (Fig. 5E). Another control group with IEMP/BMSC implant but without ultrasonic treatment also yielded an insignificant impact on days 7 and 28 (fig. S13). This evidence demonstrated that sole electrical stimulation or the BMSC delivery was not sufficient to maximize the results. In contrast, the IEMP/BMSC-treated group exhibited improved myocardial preservation thanks to the integration of stem cells and piezoelectricity. The Masson’s trichrome staining on day 28 also showed significantly thicker anterior wall tissue and less area of fibrosis formation compared to the PVDF and IEMP groups (Fig. 5F).

**Fig. 6.**
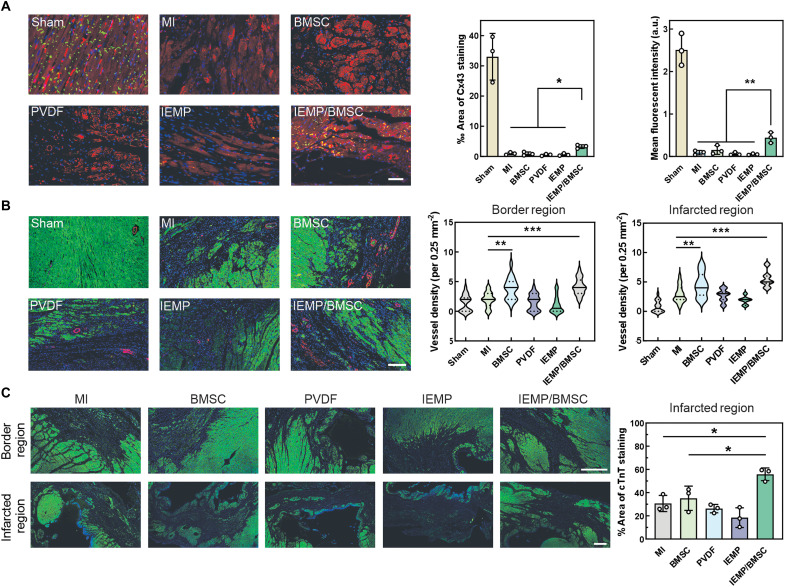
Improved connection and survival of cardiomyocytes by IEMP-mediated stem cell therapy. (**A**) The representative immunofluorescent staining of Cx43 (green), α-actinin (red), and 4′,6-diamidino-2-phenylindole (DAPI, blue). The quantitative analysis of ‰ area and mean fluorescent intensity of Cx43 staining were based on the immunofluorescent staining. Scale bar, 50 μm. *n* = 6. (**B**) Representative immunofluorescent staining of α–smooth muscle actin (α-SMA) (red), α-actinin (green), and DAPI (blue). Scale bar, 200 μm. Quantification of α-SMA^+^ arterioles was based on immunofluorescence staining in the border and infarcted regions. Scale bar, 200 μm. *n* = 18. (**C**) Representative immunofluorescent staining of cTnT (green) and DAPI (blue). Quantifications of cTnT coverage in the infarcted region were based on immunofluorescent images. Scale bar, 200 μm. Data are means ± SD *P < 0.05; **P < 0.01; ***P < 0.001.

Furthermore, the visualization of connexin 43 (Cx43) revealed a higher expression of gap junction protein in the remaining cardiomyocytes within the infarcted region of the IEMP/BMSC group compared to other treatments (Fig. 6A), suggesting an enhanced electrical coupling and metabolic communication within this area. In addition, the number of blood vessels was also elevated in both the BMSC and IEMP/BMSC groups among the border and infarcted regions (Fig. 6B). Notably, in the sham group, which was only performed open-heart surgery, their angiogenesis was minimal as healthy cardiac tissue typically lacks the biological stimulus for neovascularization and contains only a limited number of arterioles. The self-activating recovery of injured cardiac tissue appeared less effective compared to that boosted by the adoptive BMSCs. The remaining cardiomyocytes that were supported by the angiogenic area at the border and infarcted regions were also quantified by the cardiac troponin T (cTnT) marker (Fig. 6C). The fragmented but more frequent area of alive cardiomyocytes contributed to a higher overall coverage in the infarcted region, explaining the better performances in wall thickness and left ventricle functions.

## DISCUSSION

The IHD is presently the leading cause of death worldwide, resulting in millions of deaths per year (*48*). Precautions in behavior, weight control, and blood pressure were recommended to prevent the occurrence of IHD. However, limited medical operations or successful recovery were available for patients (*49*). Stem cell therapy that promised to replace or recover the damaged cardiomyocytes, which were traditionally assumed unable to rejuvenate, revolutionized heart regeneration (*50*, *51*). The pursuit of stem cells as a universal replacement of dysfunctional somatic cells has already witnessed decades of clinical application of hematopoietic progenitor cells for hematopoietic and immunologic reconstitution. In recent years, the Food and Drug Administration granted the clearance for several clinical trials of investigational new drug applications based on induced pluripotent stem cells (iPSCs) for photoreceptor disease, Parkinson’s disease, and reproductive medicine. In December 2024, the first mesenchymal stromal cell therapy for steroid-refractory acute graft-versus-host disease was approved. Nevertheless, the undesired potency of stem cells, low engraftment of transplanted cells, and highly complex disease situations complicated the clinical translation.

Multiple strategies have been explored to address the challenges of stem cell–based cardiac therapy, including modulation of the hyperinflammatory microenvironment, enhancement of stem cell function, and precise coordination of cell delivery. Preclinical studies have shown that repeated stem cell administrations yielded superior outcomes compared to a single dose, suggesting that sustained cell presence and long-term functionality might help prevent rehospitalization (*52*). A recent study reported a collagen patch embedded with iPSC-derived cells, which remained engrafted on the left ventricular wall for over 6 months and significantly alleviated ischemia-reperfusion injury in macaques (*53*). In our study, we developed an implantable electroactive device capable of encapsulating a large quantity of BMSCs for targeted and sustained delivery to the heart with high cell viability and functionality. This platform enabled effective cell-cell communication between BMSCs and cardiomyocytes, and electrical stimulation further enhanced BMSC paracrine effects while minimizing tissue damage. In a rat MI model, the IEMP device facilitated prolonged retention of BMSCs at the anterior wall of the left ventricle. The electrically potentiated BMSCs served as an endurable paracrine source and suppressed the phenotypes of inflammatory monocytes in the early stage. As a result, the heart exhibited reduced fibrotic remodeling and improved cardiomyocyte survival. In addition to the BMSC-boosting effects, the inclusion of electricity in heart therapy has already demonstrated efficacy in the self-powering nanogenerator (*54*–*57*) to harness the beating rhythm to compensate the heart functional loss. An integrated self-powered piezoelectric generator in the future could replace the necessity of ultrasonic actuation. A feasible piezoelectric or ultrasonic strategy also potentiated multiple tissue engineering innovations in surgery and brain recovery (*58*, *59*). A holistic strategy to achieve a comprehensive cell delivery strategy that not only preserves cell viability and therapeutic efficacy but also promotes dynamic cross-talk between transplanted and host cells could offer a promising solution to the persistent limitations of current cell therapies (*60*, *61*).

## MATERIALS AND METHODS

### Materials

The materials used here are as follows: polylactic acid (*M*_w_ ~ 110,000, particle size 3 mm, Rhawn), poly(l-lactide) (ester-terminated, catalog no. P299052, Aladdin), PVDF-TrFE (80/20 mol %, PolyK Technologies Co. Ltd.), and PVA (degree of hydrolysis: 98.0 to 99.0 mol %, Aladdin),

### Fabrication of IEMP

All molds were designed by SOLIDWORKS 2022 and printed by nanoArch S130 3D printer (Boston Micro Fabrication Co. Ltd.) with 10-μm resolution in *z* axis and HTL resin. The printed molds underwent three-step posttreatments, immersing in isopropanol for 12 hours, drying in 90°C oven for 4 hours, and ultraviolet (405 nm) recuring for at least 30 min with the light projected to each facet. To obtain PDMS molds, SYLGARD184 Silicone was poured into the posttreated molds and cured in 60°C oven for 1 hour. To fabricate IEMP, 50 mg of PLLA was placed on the bottom mold and incubated in 235°C vacuum oven (DP33C, Yamato Scientific Co. Ltd.) for 30 min to fully liquefy. A −0.1-Mpa vacuum was maintained for 20 min to remove the excessive air before cooling down the mold at room temperature. Later, the top mold was placed onto the solidified PLLA and aligned with the bottom mold tip by tip with the help of the transparency of PDMS. Next, the sandwich molds returned to 235°C, −0.1-Mpa vacuum oven and the convex parts of top mold would sink into the concave parts of the bottom mold. The perforated structure and MN would therefore be obtained after recooling the sandwich molds to room temperature. Other shapes or types of material followed the same processes as mentioned. For PVA IEMP, 5% of PVA 1788 (Aladdin) solution was dipped onto the bottom mold, vacuumed, and then evaporated in the oven two times to obtain the IEMP.

### Supercooling molding of IEMP

To improve the piezoelectricity and crystallinity of PLLA, the as-generated IEMP MN in “sandwich” molds incubating in 235°C oven was swiftly transferred to 157°C (DZF-6032, Shanghai Yiheng Co. Ltd.) oven without temperature drop. After the designated time, the sandwich molds were transferred to room temperature and allowed to cool down.

### Measurement of piezoelectricity

The posttreated PLLA film or IEMP was sandwiched into a device by two copper tapes as electrodes, and then the device was sealed with a thin layer of SYLGARD184 to avoid external interference. For the film measurement, the distances between two copper electrodes were around 1000 μm unless the film thickness was intentionally manipulated. The overlapping area of the two tapes was around 1 cm^2^. For the IEMP, one electrode stuck to the base of the IEMP with a contact area of around 63.6 mm^2^. Also, another electrode was tightly stuck to the tips of the needle patch. The total overlapping area of the two tapes was around 1 cm^2^. For the in vitro setting, the sealed device was placed on a metal platform that was fully covered by packaging tape. The probe of the ultrasonic machine (Moreanva, India, 1 MHz) was placed around 1 mm above the device, where the interval was fully occupied by ultrasonic gel. The probe and platform were held still throughout the experiment. The two copper tapes were connected to the oscilloscope (RIGOL DS1054) with an additional 680-Ω resistance to exclude the interference of the ambiance. For the ex vivo experiments in pork, the sealed device was placed between the muscle layer and the adipose layer, around 2 cm beneath the skin. The probe of the ultrasonic machine was placed 1 mm above the pork skin, and the interval was filled with ultrasonic gel. As for the measurement of piezoelectric coefficient, a PolyK Quasi Static Piezoelectric Meter was directly used to measure.

### Fabrication of PVDF-TrFE film

Ten % PVDF-TrFE was dissolved in 1,4-dioxane, and 5 ml of the liquid was evaporated in a 10-cm glassy petri dish for 2 days in a hood. The generated flat film around 200 μm thick will be electretized by 12 kV for 120 min at room temperature.

### X-ray powder diffraction

Data were collected at room temperature on Bruker D8 Discover diffractometer with parafocusing Bragg-Brentano geometry and 1D LynxEye XE-T detector using a Cu Kα radiation (λ = 1.5418 Å), and operated at 42 kV and 100 mA. The scan 2θ range was from 5° to 60° with a step size of 0.02° and a counting time of 0.2 s per step.

### Differential scanning calorimetry

Samples were analyzed by Thermal Analysis System DSC 3^+^ under a nitrogen atmosphere. Each sample weighed about 2 mg. A heating rate of 10°C min^−1^ in the range of 40° to 200°C was selected.

### Mechanical test of PLLA

The elastic modulus and the yield point (force per patch) were calculated by a universal testing machine (Xinsansi, C61.103) with stress-strain curve. The tested PLLA block in the size of 4 mm by 1 by 8 mm was clamped by two pliers moving at a speed of 1 mm min^−1^ until breakage. The IEMP patch was placed on a stationary plate, and the opposite plate was descending at the speed of 0.5 mm min^−1^. All data were collected at 120 Hz.

### Cell extraction and culture

BMSCs were harvested from 1-week-old Sprague-Dawley rats by rinsing both tibia and femur cavity as previously described (*62*). The flushed marrow was collected in complete DMEM culture [10% fetal bovine serum (FBS) and 1% streptomycin-penicillin] and passed through a 70-μm filter to remove the tissue debris. Later, the bone marrow cells were cultured in 10-cm cell-culture dishes with complete DMEM at 37°C, 5% carbon dioxide for 48 hours. The nonadherent cells were discarded, and the adherent cells were passaged for four passages before use. Neonatal rat cardiomyocytes were extracted by scissoring the left ventricle into 1-mm^3^ fragments. The tissue fragments were digested by a trypsin/collagenase type II mixture for 10 min and 10 times. The collected cell suspension was placed in cell culture flasks for 30 min and two times to remove the excessive fibroblasts. The suspended cardiomyocytes were collected through centrifugation and configured to 120,000 cm^−2^ in culture dishes.

### BMSC loading into IEMP

The IEMP part and the lid part were assembled under the microscope in a sterilized environment. An electric iron was used to tightly seal the interstice between the two parts, only leaving a tiny hole for 32G needle tip to insert. The BMSC was resuspended as 3 × 10^6^ ml^−1^ in 4% gelatin DMEM medium (without FBS) at 37°C, and 50 μl of which was injected into the assembly through the tiny hole. The hole was later sealed by an electric iron. The assembly was placed at 4°C for around 60 min before application to the animal.

### Cell release behavior of BMSC

Complete DMEM was used as the medium for in vitro release. Specifically, 2 ml of DMEM was placed in a 24-well plate, and BMSC-loaded IEMP was placed floating on the medium. Two hundred microliters of the culture medium was extracted at the designated time points, and the same amount of medium was supplemented. The extracted cells were counted by a cell counter (Countess 3) or hemocytometer. For releasing into the GelMA hydrogel, 200 μl of GelMA solution was injected into the confocal dish. An empty IEMP was placed on the GelMA solution before applying ultraviolet radiation (405 nm) to cross-link the GelMA hydrogel and casted a shape that matched the curvature of the IEMP assembly. The as-generated GelMA gel was freeze-dried into a solid and submerged into 200 μl of complete DMEM culture to recover to a hydrogel. Later, the IEMP assembly loaded with 1.5 × 10^5^ BMSC inside was placed onto the hydrogel, and 100 μl of DMEM was used to moisten the interface between IEMP and GelMA. Confocal images (by Nikon AX) were taken at the designated time points.

### The in vitro setting of electric stimulation

The IEMP device (with or without BMSC loaded) was placed into the culture dishes of routinely cultured cells. The ultrasonic probe was applied 5 mm underneath the base of the culture plate, and the interspace was filled with ultrasonic gel. For measuring the paracrine effects after piezoelectric stimulation, blank IEMP was placed in the medium with 1.5 × 10^5^ BMSCs seeded in a well plate. BMSCs were collected 3 or 7 days later. An ultrasonic therapeutic machine was applied every 8 hours (1.0 W/cm^2^ for 3 min) for three times. For measuring mRNA expression of cardiomyocytes, the IEMP/BMSC assembly was coincubated with 2 × 10^6^ cardiomyocytes four days after extraction, and an ultrasonic therapeutic machine was applied every 8 hours (1.0 W/cm^2^ for 3 min) for three times. Cardiomyocytes were collected 24 hours later. For direct current stimulation, ac was generated by RIGOL DG1022, and all voltages were set at 5000 Hz.

### Ischemic challenging for cardiomyocytes

The extracted cardiomyocytes were maintained for 4 days before being transferred to the incubator with AnaeroPack. Various amounts of BMSCs were added to 5 × 10^5^ cardiomyocytes through transwells. The apoptosis rate of cardiomyocytes was determined through TUNEL (terminal deoxynucleotidyl transferase deoxyuridine triphosphate nick end labeling) assay 24 hours after coincubation.

### Angiogenesis assay

Human umbilical vein endothelial cells were seeded at the concentration of 5 × 10^5^ cells per milliliter of DMEM culture onto the cross-linked Matrigel and were allowed for 1 hour to settle. The IEMP/BMSC embedding 1.5 × 10^5^ BMSC or blank IEMP was placed floating on the surface of the culture medium and waited for 4 hours. Later, the cells were observed by fluorescent cytometry after incubating with 5 μM Calcein AM for 10 min.

### Animal experiments

All animal experiments were approved by Zhejiang University Laboratory Animal Center (ZJU20240392). Acute MI was established on 6- to 7-week-old female Sprague-Dawley rats (220 ± 20 g). Two % isoflurane inhalation was applied to anesthetize the animals, and then they were intubated and ventilated with a respirator with extra oxygen. The chest was depilated and disinfected with povidone-iodine solution. The thoracotomy was conducted at the fourth intercostal space at its supine position. The pericardium was first removed with tweezers, and the left anterior descending artery was ligated at around 2 mm from its origin between the left atrium and pulmonary artery conus with a 6-0 suture. The successful ligation will witness the color of the anterior wall of the left ventricle changing from deep red to pale red. Then, the rats randomly received the following treatments: Sham group: Four points of the anterior wall of left ventricle were punctured with C-22 needle. IEMP/BMSC group: Four edges of the IEMP were sutured on the ischemic area with 6-0 suture and C-22 needle; each cavity of the tip carried 1.25 × 10^4^ BMSCs (1.5 × 10^5^ per patch). IEMP group: Four edges of the IEMP were sutured in the same manner. PVDF group: A PVDF-TrFE film with the same size as IEMP (9 mm in diameter) was sutured at four edges on the ischemic area in the same manner. BMSC group: BMSCs (1.5 × 10^5^) were intracardially injected into two locations of the ischemic area. No ultrasound: the same procedure as the IEMP/BMSC group but without further ultrasonic treatment. Then, the chest was closed with 5-0 silk sutures, and the surviving animals were maintained on standard rat chow. The ultrasound was applied on the left chest for 72 hours or extended time if stated elsewhere. For live imaging of DiR (1,1'-dioctadecyl-3,3,3',3'-tetramethylindotricarbocyanine iodide)-labeled BMSCs, 5 μM DiR was incubated with BMSCs for 10 min before the same amount of BMSC and IEMP/BMSC were applied to rats.

### Echocardiography

Rats were anesthetized by 2% isoflurane inhalation at supine position, and the M-mode echocardiographic data were collected on days 7 and 28 by Vevo 2100 ultrasound equipment (Visual Sonics, Canada). The echocardiographic parameters, including left ventricle (LV) dimensions, wall thicknesses at the diastolic and systolic states, LV ejection fraction, and LV fractional shortening, were automatically measured and calculated by the machine.

### The histological and immunofluorescent analysis

Hearts were collected at the indicated time points and submerged in 4% polyoxymethylene, sliding into paraffin section and staining with H&E stain or Masson’s trichrome stain. For immunofluorescent slides, the following primary antibodies were purchased from Proteintech and Abcam: Cx43 polyclonal antibody (26980-1-AP), alpha actinin monoclonal antibody (66895-1-Ig and 11313-2-AP), cTnT polyclonal antibody (15513-1-AP), smooth muscle actin–specific monoclonal antibody (67735-1-Ig), anti-CD86 antibody (ab220188), anti-CD206 antibody (ab64693), goat anti-rabbit immunoglobulin G (IgG) HL (ab150077), anti-mannose receptor antibody (ab64693), and goat anti-mouse lgG HL (ab150116). The wall thickness of the left ventricle was measured by dividing the infarcted region into five sections and calculating the average thickness from these five by Olympus Image Viewer. The percentage of fibrosis area, calculation of the area percentages of Cx43 and cTnT staining, and corresponding mean fluorescent intensity were conducted by ImageJ. Six selected fields of view were evenly sampled from the infarcted/border region of each heart section. The quantification of blood vessels was manually calculated by viewing the whole border region.

### Fluorescence-activated cell sorting of monocytes

Phycoerythrin anti-CD43 (W3/13, BioLegend) and fluorescein isothiocyanate anti-His48 (HIS48, eBioscience) were purchased as instructed (*63*) and titrated according to the guidance from the manufacturer. Briefly, for the in vivo experiment, the anterior wall of left ventricle was harvested at the indicated time and digested with collagenase II (400 U/ml) and deoxyribonuclease I (48 U/ml). A 42% isotonic Percoll solution was used to concentrate the immune cells in single-cell suspension (*64*). For the in vitro experiment, monocyte was collected by rinsing both the tibia and femur cavity and stimulated with macrophage colony-stimulating factor for 4 days. At day 4, IEMP was placed into the medium, and everyday ultrasonication for 3 min (0.5 W/cm^2^) was applied until day 7 to analyze. All samples were analyzed by Beckman CytoFlex S and FlowJo software.

### Paracrine analysis

The protein levels were measured by enzyme-linked immunosorbent assays as the manufacturer’s instructions (Multi Sciences Co. Ltd). The culture medium was directly collected, and measured. The mRNA levels were determined by polymerase chain reaction (PCR). All cultured cell samples were rinsed with fresh phosphate-buffered saline solution three times before being lysed with TRIzol reagent. The total mRNA was extracted to synthesize cDNA by Evo M-ML RT kit (Accurate Biotechnology Co. Ltd) as the manufacturer instructed. The relative expression of mRNA was determined through SYBR Green Pro Taq HS quantitative PCR kit as instructed. All primers were listed in table S1.

### Statistical analysis

All results are presented as means ± SD unless specified elsewhere. Statistical analysis was evaluated by GraphPad Prism 9.0.0 with analysis of variance (ANOVA) or Student’s *t* test. The differences among groups were considered statistically significant when *P < 0.05; **P < 0.01; ***P < 0.001.
