# Supplementary material for: Electroactive microneedle augmented stem cell therapy in myocardial infarction
Source: Sci Adv. 2026 May 6;12(19):eaeb4840. doi: 10.1126/sciadv.aeb4840 (PMC13148333; doi:10.1126/sciadv.aeb4840)
Supplement: Supplementary file 1 — Figs. S1 to S13 Tables S1 [file sciadv.aeb4840_sm.pdf]

Science Advances
AAAS

Supplementary Materials for

Electroactive microneedle augmented stem cell therapy in myocardial infarction

Wentao Zhang *et al.*

Corresponding author: Yuqi Zhang, yqzhang21@zju.edu.cn; Zhuxiao Gu, gzx@nju.edu.cn;
Zhen Gu, guzhen@zju.edu.cn

Sci. Adv. **12**, eaeb4840 (2026)
DOI: 10.1126/sciadv.aeb4840

This PDF file includes:

Figs. S1 to S13
Table S1

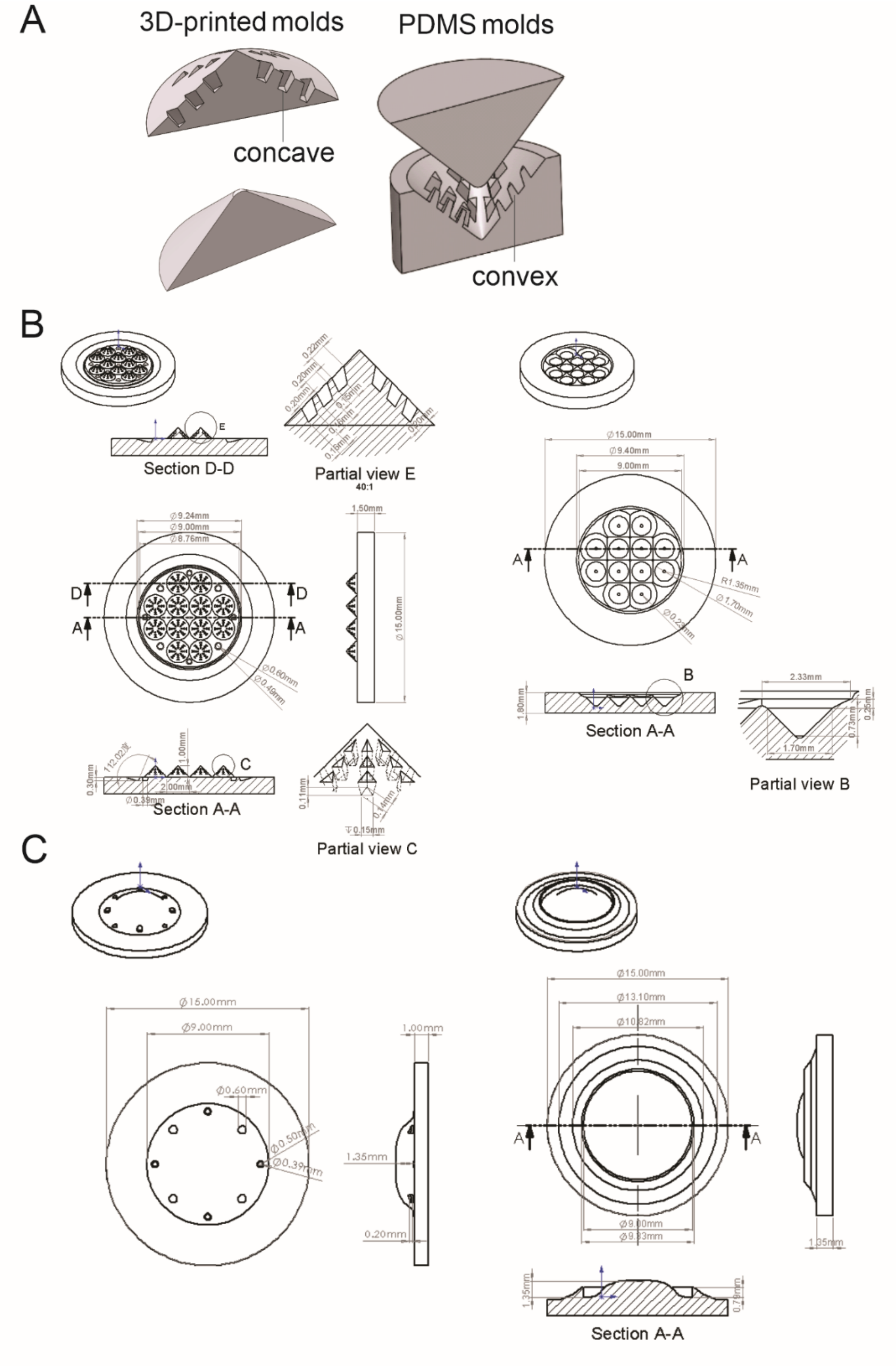

Supplementary Figure 1. **Designs of 3D printed molds.** A, The illustration of the alignment and perforation process of PDMS molds. B, The designs of two 3D-printed molds to fabricate the IEMP. C, The designs of two 3D-printed molds to fabricate the base.

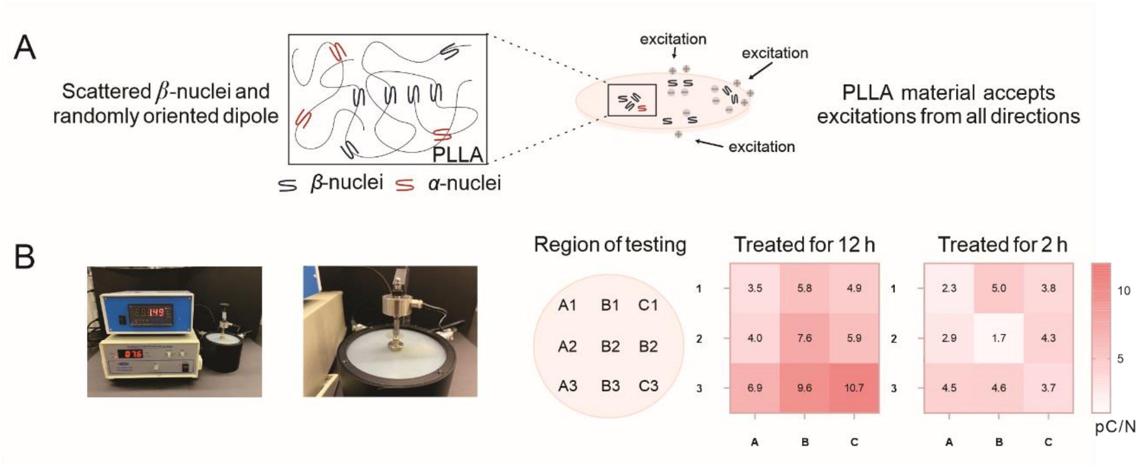

Supplementary Figure 2. **Demonstration of supercooling-induced piezoelectricity.** A, the graphic scheme to explain the scattered *β*-nuclei and randomly oriented dipole after supercooling. B, the measurement of the piezoelectric coefficient at different regions.

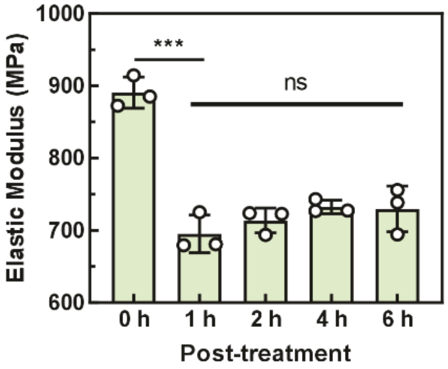

Supplementary Figure 3. **Measurement of the elastic modulus.** The measurements of the elastic modulus of the PLLA block after different post-treatment times. n = 3. Data are means ± s.d. *P<0.05; **P<0.01; ***P<0.001.

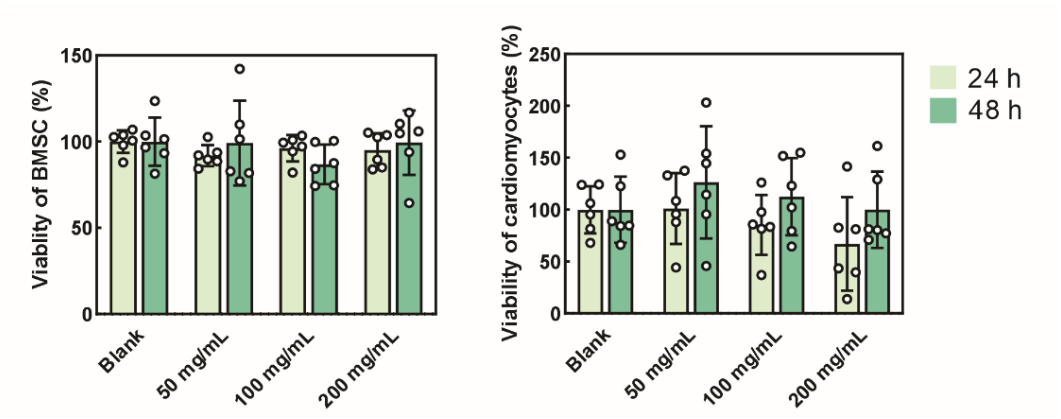

Supplementary Figure 4. **Biocompatibility of PLLA.** The viability of BMSCs and cardiomyocytes after incubating with as-prepared PLLA (50, 100, 200 mg/mL) for 24 and 48 h. $n = 6$. Data are means ± s.d..

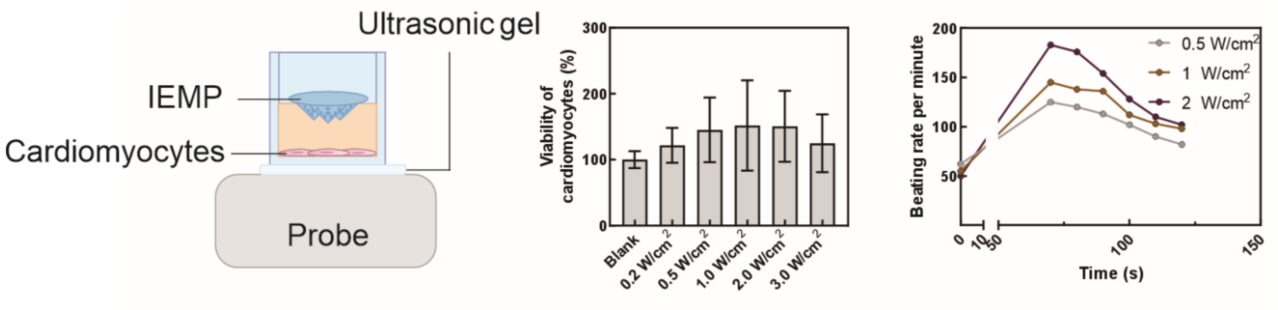

Supplementary Figure 5. **Influences of piezoelectricity to cardiomyocytes.** The *in vitro* setting of measuring the responses of cardiomyocytes to piezoelectricity. The cell viability was tested 24 hours after the stimulation for 2 min, and the beating rate was tested immediately after the stimulation for 1 min. n = 4 for viability. The beating rate was calculated at each time point based on the performance in the recent 10 seconds by observing the same batch of cardiomyocytes. Data are means ± s.d..

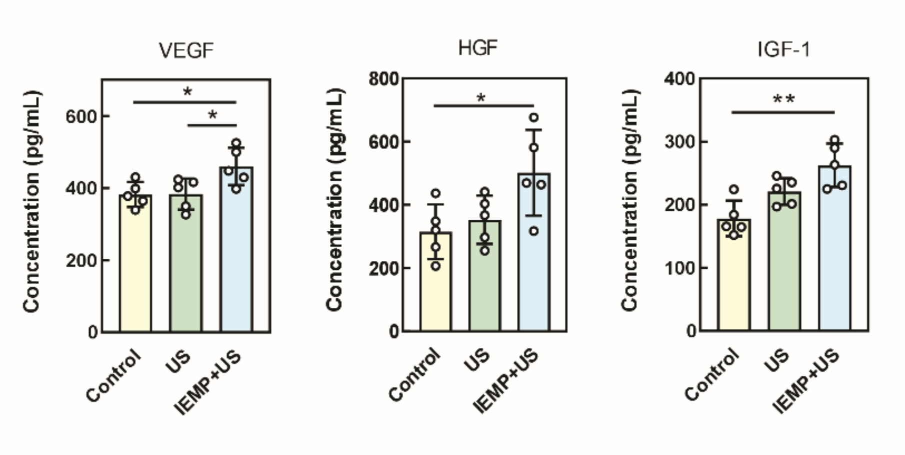

Supplementary Figure 6. **Paracrine analysis of BMSCs on protein level.** The protein expressions of VEGF, HGF, and IGF-1 in BMSCs after stimulation with ultrasound only or IEMP+ultrasound at day 7. *n* = 5. Data are means ± s.d.. *P<0.05; **P<0.01; ***P<0.001.

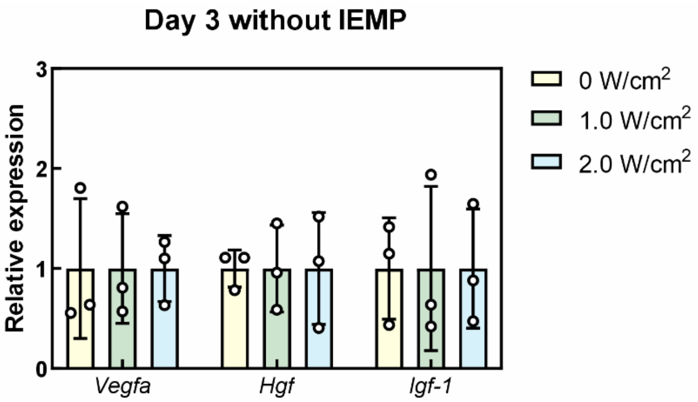

Supplementary Figure 7. **Ultrasound effects on the BMSCs in paracrine transcription.** The relative expressions of *Vegfa*, *Hgf,* and *Igf-1* compared to *Gapdh* after stimulation of ultrasound but without IEMP for 3 days. n = 3. Data are means ± s.d..

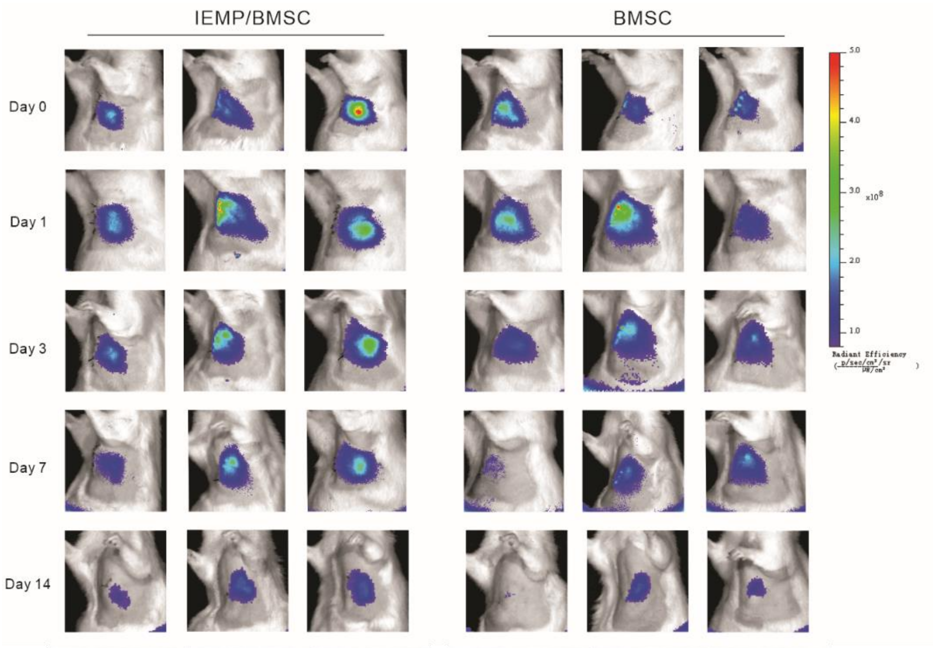

Supplementary Figure 8. **The retention of delivered BMSCs**. Representative *in vivo* fluorescence images showing the retention of DiR-labeled BMSCs in rats at different time points. IEMP/BMSC was administered by the IEMP device at the anterior wall of left ventricle, and BMSCs were administered by intracardiac injection.

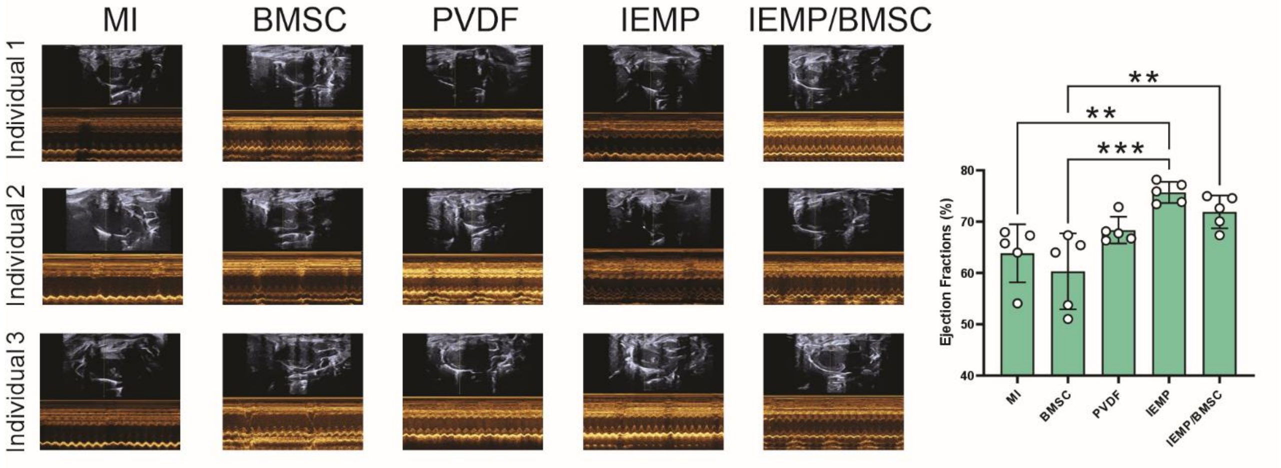

Supplementary Figure 9. **Echocardiography of hearts in different groups on day 3.** Representative M-mode echocardiography images of different groups on day 3 and quantitative analysis of LV ejection fraction according to the echocardiography. *n* = 5. Data are means ± s.d. **P*<0.05; ***P*<0.01; ****P*<0.001.

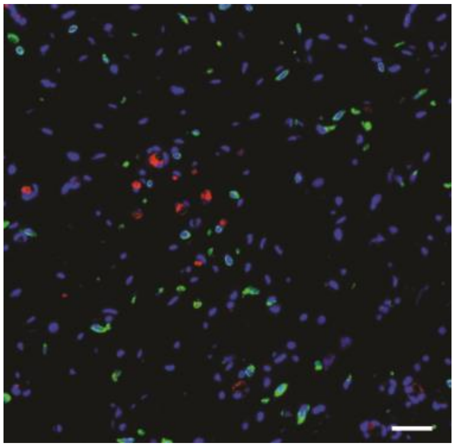

Supplementary Figure 10. **Immunofluorescent analysis of macrophages phenotypes.** The fluorescent image of the heart section on day 2. The red signal was likely false-positive because it did not surround the nucleus signal. Blue, DAPI. Red, anti-CD86. Green, anti-CD206. Scale bar, 50 µm.

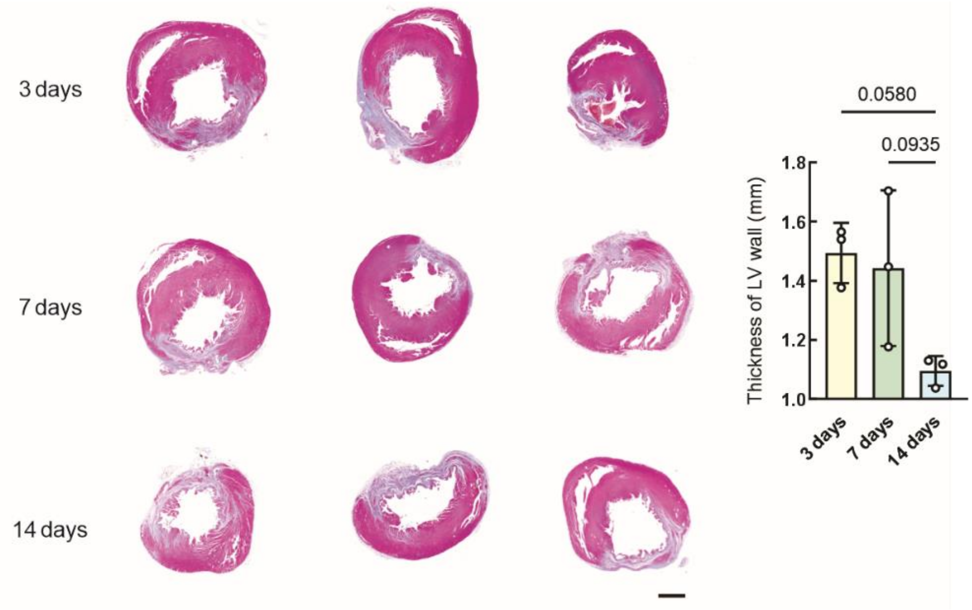

Supplementary Figure 11. **Histological analysis of the heart section with varied piezoelectric stimulations.** Masson's trichrome staining of heart after different time periods of piezoelectric stimulation. 3 and 7 days indicated intermittent ultrasonic treatment every 8 hours, and 14 days indicated daily ultrasonic treatment for 14 days. 3 min each time. The quantitative analysis was based on the images. Scale bar, 2 mm. *n* = 3. Data are means ± s.d..

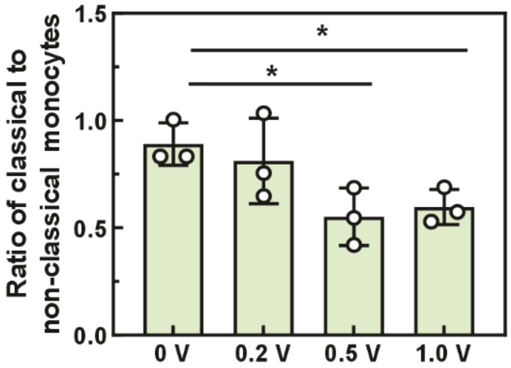

Supplementary Figure 12. **Phenotype analysis of monocytes.** *In vitro* analysis of the ratio of classical monocytes ($CD43_{lo}His48_{hi}$) to the non-classical monocytes ($CD43_{hi}His48_{int\text{-}lo}$) based on the FACS results. n = 3. Data are means ± s.d. *P<0.05.

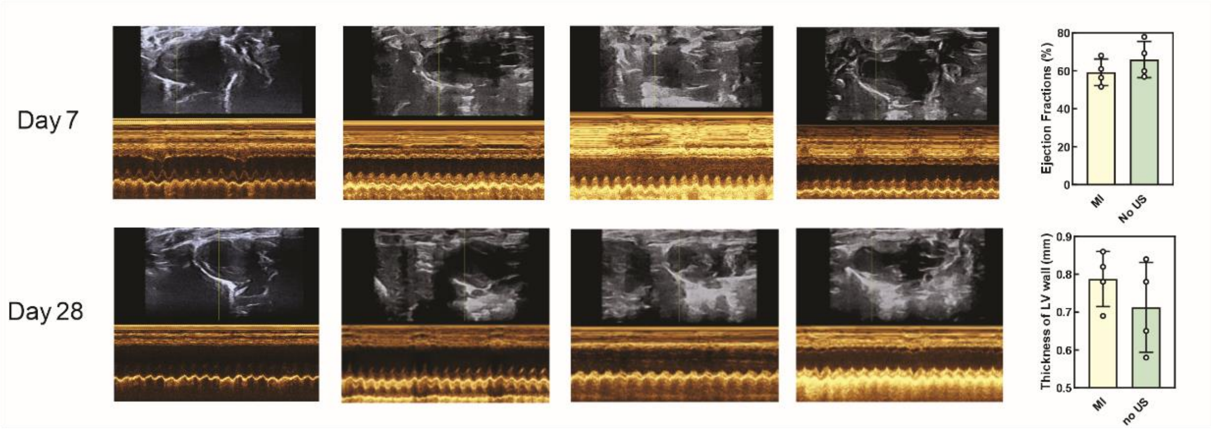

Supplementary Figure 13. **Echocardiography of hearts without ultrasonic treatment.** M-mode echocardiography images of the rat with IEMP/BMSC but without ultrasonic treatment (no US) on day 7 and day 28. Quantitative analysis of LV ejection fraction and thickness of LV wall were based on the echocardiography. n = 4. Data are means ± s.d..

Supplementary Table 1. Primer(5'-3') table for qRT-PCR

	Forward primer	Reverse primer
Pi3k	CATGGATGCTTTGCAGGGTTT	CCAGATGTTCTCCATGATTCGGA
Pten	AGACCATAACCCACCACAGC	TACACCAGTCCGTCCTTTCC
Akt1	TAGGCATCCCTTCCTTACAG	GCCCGAAGTCCGTTATCT
Anp	CTTCGGGGGTAGGATTGAC	CTTGGGATCTTTTGCGATCT
Rhoa	CATCCCAGAAAAGTGGACTCCA	CCTTGTGTGCTCATCATTCCG
Vegfa	AGTCCCATGAAGTGATCAAGTTCA	CACTCCAGGGCTTCATCGTT
Igf1	GTGGATGCTCTTCAGTTCGTGTG	GAAGCAACACTCATCCACAATGCC
Hgf	GTCCTGAAGGCTCAGACTTGGT	CCAGCCGTAAATACTGCAAGTGG
Actin	AGAGGGAAATCGTGCGTGAC	AGGAAGGAAGGCTGGAAGAGA
Gapdh	CTCATGACCACAGTCCATGC	TTCAGCTCTGGGATGACCTT
